# Estimation of Sensitivity and Specificity of Bacteriology, Histopathology and PCR for the Confirmatory Diagnosis of Bovine Tuberculosis Using Latent Class Analysis

**DOI:** 10.1371/journal.pone.0090334

**Published:** 2014-03-13

**Authors:** Aurélie Courcoul, Jean-Louis Moyen, Laure Brugère, Sandy Faye, Sylvie Hénault, Hélène Gares, Maria-Laura Boschiroli

**Affiliations:** 1 Epidemiology Unit, Paris-Est University, Anses, Laboratory for Animal Health, Maisons-Alfort, France; 2 Regional Analysis and Research Laboratory of Dordogne (LDAR24), Coulounieix-Chamiers, France; 3 Bovine Tuberculosis Reference Laboratory, Paris-Est University, Anses, Laboratory for Animal Health, Bacterial Zoonoses Unit, Maisons-Alfort, France; Auburn University, United States of America

## Abstract

Bacteriology and histopathology are the most commonly used tests used for official confirmatory diagnosis of bovine tuberculosis (bTB) in cattle in most countries. PCR is also being used increasingly because it allows a fast diagnosis. This test could be applied as a supplement to or replacement for current bTB confirmatory diagnostic tests but its characteristics have first to be evaluated. The aim of this study was to estimate and compare sensitivities and specificities of bacteriology, histopathology and PCR under French field conditions, in the absence of a gold standard using latent class analysis. The studied population consisted of 5,211 animals from which samples were subjected to bacteriology and PCR (LSI VetMAX™ *Mycobacterium tuberculosis* Complex PCR Kit, Life Technologies) as their herd of origin was either suspected or confirmed infected with bTB or because bTB-like lesions were detected during slaughterhouse inspection. Samples from 697 of these animals (all with bTB-like lesions) were subjected to histopathology. Bayesian models were developed, allowing for dependence between bacteriology and PCR, while assuming independence from histopathology. The sensitivity of PCR was higher than that of bacteriology (on average 87.7% [82.5–92.3%] versus 78.1% [72.9–82.8%]) while specificity of both tests was very good (on average 97.0% for PCR [94.3–99.0%] and 99.1% for bacteriology [97.1–100.0%]). Histopathology was at least as sensitive as PCR (on average 93.6% [89.9–96.9%]) but less specific than the two other tests (on average 83.3% [78.7–87.6%]). These results suggest that PCR has the potential to replace bacteriology to confirm bTB in samples submitted from suspect cattle.

## Introduction

Bovine tuberculosis (bTB), caused principally by *Mycobacterium bovis*, is an on-going issue in several parts of Europe. Although eradication and control programs have been in place for many decades, the prevalence of bTB is currently increasing both in non-bTB-free and in officially bTB-free EU countries [1]. In this context, detection and confirmation of bTB infection have to be fast and reliable to ensure efficient surveillance and control programs. Knowledge about accuracy of the tests used to detect and confirm infection seems, then, essential. Detailed information on the characteristics of tests used for bTB detection (such as skin tests or γ-interferon tests) is available [2]–[4]. However, the sensitivity and specificity of tests used for bTB confirmatory diagnosis are far less known. In the EU, the presence of *M. bovis* in a sample may be officially demonstrated by histopathology and confirmed by bacterial culture. Histopathology is less specific than bacterial culture: Varello et al. [5] demonstrated that the average specificity of histological methods was 92.3% when compared with bacteriology as reference test. This often requires, when a sample is histopathology positive, confirmation of the infection by bacteriology. In France, cattle herds are subjected to movement restrictions if they present positive screening tests results. In order to lift the restriction, either reactors have to be tested negative to a second skin test or, if they are culled, they have to be tested negative to confirmatory tests (in this case, the herd has sometimes also to be tested negative to a second skin test to lift the restriction). Bacteriology, which takes at least three weeks but more often up to three months, has thus important constraints on the field. Besides, its sensitivity is not perfect, around 85% in samples with visible lesions (European Union Reference Laboratory for Bovine Tuberculosis, personal communication). In this setting, an alternative to obtain a faster infection confirmation or invalidation could be PCR. When infection is confirmed, it would allow a responsive herd management (i.e. whole or partial herd culling, tracing of contact herds, investigation of surrounding wildlife, etc.). When infection is not confirmed, movement restrictions could quickly be lifted.

The LSI VetMAX™ *Mycobacterium tuberculosis* Complex PCR Kit, distributed by Life Technologies, enables the detection of *M. tuberculosis* complex (MTBC) organisms. It detects the IS*6110* specific insertion sequence found in the genome of all MTBC members. This PCR may be used as a supplement to or replacement for current bTB confirmatory diagnostic tests (i.e. bacterial culture and histopathology) but, to our knowledge, it has not yet been evaluated from samples obtained as part of routine bTB surveillance and control programs, which seems a pre-requisite for a sensible use.

The performance of a diagnostic test is classically evaluated against a perfect test defined as a gold standard. As previously stated, none of the tests used to confirm bTB infection has 100% sensitivity and 100% specificity and can thus be considered as a gold standard. When a reference test is unavailable, a Bayesian formulation of the latent class analysis can be an option to assess the sensitivity and specificity of diagnostic tests [6]. The disease status of the tested individuals is designated “latent” (existing but not presently evident or realized) and none of the tests are considered a reference test. In latent class analysis, three assumptions known as the Hui-Walter paradigm are generally made [7]: (1) two or more populations with differing prevalences are required; (2) the sensitivity and specificity of the tests are constant across all populations; and (3) the tests are conditionally independent. For tests based on the same biological basis, this latter assumption can be relaxed [2], [8], [9]. In our case, it seems reasonable to assume that bacteriology and PCR are dependant tests as they both detect the mycobacterium directly.

The aim of this study was therefore to estimate, using latent class analysis and accounting for conditional dependence between bacterial culture and PCR, the sensitivity and the specificity of the diagnostic tests (bacterial culture, histopathology and PCR) carried out in France on lymph nodes in order to confirm or invalidate bTB diagnosis on suspect animals (e.g. animals positive to skin tests, animals with macroscopic bTB-like lesions or animals from infected herds).

## Materials and Methods

### 1. Ethical Statement

bTB is a notifiable disease for which there are control and surveillance campaigns in France. Official methods for diagnosis of this disease are culture, PCR and histopathology. Therefore, all the samples included in this study are issued from animals analyzed within an official context. No purpose killing of animals was performed for this study. All samplings were in complete agreement with national and European regulations. No ethical approval was necessary.

### 2. Sample Collection and Population Stratification

The source population was the animals whose samples were submitted to the “Laboratoire d’Analyse et de Recherche de Dordogne” (LDAR 24) for confirmation of *M. bovis* infection between 2008 and 2012. These animals were analyzed because (i) they presented non negative skin tests and/or γ-interferon tests results and were slaughtered for diagnostic purposes, (ii) they were slaughtered as a result of the total or partial slaughter of their herd of origin after confirmation of the herd’s infection, or (iii) they presented macroscopic bTB-like lesions at routine abattoir inspection. Samples from the totality of animals submitted to the laboratory for bTB confirmatory diagnosis between 2008 and 2012, except for 30 animals for which PCR and bacteriology results or herd of origin were not available, were retrospectively enrolled in the study. The study sample included 5,211 individuals from 1,325 French cattle herds in 51 departments (over 96 in metropolitan France).

Tracheobronchial, retropharyngeal and mediastinal lymph nodes were sampled on each animal. Bacteriology and PCR were performed at the Laboratoire d’Analyse et de Recherche de Dordogne (LDAR 24). An animal was considered bacteriology positive (PCR positive respectively) if at least one of the samples was bacteriology positive (PCR positive respectively). Among these 5,211 animals, samples from 697 individuals belonging to 358 herds were also subjected to histopathology upon request either of the official veterinary services or the LDAR 24. All the animals tested using the three tests (bacterial culture, histopathology and PCR) presented macroscopic bTB-like lesions. On the contrary, the 4,514 remaining animals had no bTB-like lesions.

The herd incidence of infection over the 2000–2007 period in the cantons from where the animals originated was used to retrospectively define three populations with differing expected animal prevalences (see Figure S1). In France, a canton is a small geographical area (on average, 141 km^2^) which includes several villages. The first population (population A) comprised animals from cantons with an incidence superior to 0.25 reported cases per 100 herd-years over the 2000–2007 period. The second population (population B) comprised animals from cantons with an incidence superior to 0 but inferior or equal to 0.25 reported cases per 100 herd-years over the same period. The third population (population C) comprised animals from cantons where no bTB outbreak had been reported between 2000 and 2007.

### 3. Diagnostic Tests

#### 3.1. Preparation of the samples for PCR and culture

PCR and culture were performed on the above mentioned individual lymph nodes, presenting or not lesions at the abattoir. Lymph nodes were analyzed individually. Samples were submitted to the LDAR 24 chilled (approximately 4°C) within 48 hours after their sampling at the abattoir. Prior to PCR or culturing, 2 to 5 g of sampled tissue were crushed with a 4% H_2_SO_4_ solution to decontaminate the tissue. After 10 min, the acid was neutralized by adding a 6% NaOH solution.

#### 3.2. PCRs

A commercial kit (LSI VetMAX™ *Mycobacterium tuberculosis* Complex PCR Kit 2 wells) was used. The targeted sequence was IS*6110*, which is present in all species of the *M. tuberculosis* complex [10]. After mechanical lysis of tissue, DNA was extracted by using the QIAamp DNA mini kit (Qiagen) or by Magvet MV384 (Life Technologies) with a King Fisher KF96 automate, following the manufacturer’s instructions. Then, 5 µl of the extracted DNA was mixed with 20 µl of reaction mix and the reaction was carried out at 50°C for 2 minutes (1 cycle), followed by one cycle of 10 min at 95°C and 40 cycles of 15 s at 95°C and 1 min at 60°C. Results were interpreted as negative, positive (CT≤38) or invalidated, following the manufacturer’s recommendations and by comparison with negative and positive controls. Even if laboratory personnel were aware that sampled animals were suspect of bTB infection, they delivered PCR results as soon as they were available, without knowing bacteriology and histopathology test results.

#### 3.3. Bacteriology

Bacterial culture was performed following the protocol established by the French NRL (NF U 47–104) for isolation of *Mycobacterium bovis*. After decontamination, the supernatant was seeded on two different media: Löwestein-Jensen and Colestos [11]. All seeded media were incubated at 37°C+/−3°C for 3 months and examined every 2 weeks. The isolated *Mycobacterium tuberculosis* complex colonies were confirmed by DNA amplification as described by Hénault et al. [12] and *M. bovis* was confirmed by Luminex spoligotyping as described by Zhang et al. [13].

#### 3.4. Histopathology

When histopathology was requested by the official veterinary services, part of the samples was fixed in formalin by the LDAR 24 and sent at room temperature to one of the two accredited laboratory for histopathology (Regional Analysis Laboratory of Côte d’Armor [LDA22] and Pathologic Anatomy Laboratory of the National Veterinary School in Lyon [ENVL]). These laboratories performed hematoxylin-eosine histopathology and Ziehl Neelsen staining on each sample.

### 4. Statistical Model

Two analyses were carried out using a Bayesian formulation of the latent class model. The first analysis aimed at assessing sensitivity and specificity of bacteriology and PCR from the tests results of the 5,211 animals enrolled in the study. The second one aimed at assessing sensitivity and specificity of bacteriology, histopathology and PCR from the test results of the 697 animals subjected to the three tests (these latter animals had bTB-like lesions).

#### 4.1. First analysis

Bacteriology and PCR were not considered independent as they both detect MTBC directly. Two tests are considered to be conditionally independent when knowledge of the outcome of the first one gives no information about the outcome of the second one, conditional on the true disease status [14]. Even if PCR detects DNA whereas bacteriology detects viable bacteria, their results are expected to be dependent, relying on an animal’s true infection status. As explained by Gardner et al. [15], conditional dependence of test sensitivities occurs when the second (first) test has different sensitivities for infected animals that test-positive and for those that test-negative on the first (second) test. This was considered here to be the case for both tests sensitivities and specificities. Therefore dependence between bacteriology and PCR was accounted for. However, the correlation given infection status between these two tests was unknown. Considering a model for these two tests with three sampled populations, there were therefore nine parameters to be estimated (three prevalences, two sensitivities, two specificities and two covariances between tests). Although this model yields 9 degrees of freedom (three from each population), it lacks identifiability, i.e. the parameters cannot be estimated based on data alone [16]. For the Bayesian, identifiability is not obligatory if good prior information is available. However, in our case, we had no reliable information neither on the prevalence of infection at the animal level in populations A, B or C, nor on the characteristics of bacteriology or PCR under French field conditions. In order to ensure that the model was identifiable, the conditional covariances between bacteriology and PCR were fixed. Thus, there were only seven parameters to estimate. The model used for this first analysis was basically the same as the one used in Paul et al. [8]. We ran eight different models using covariances expressed as a proportion (i.e. 0.0, 0.1, 0.2, 0.3, 0.4, 0.5, 0.6 and 0.7) of the maximum conditional covariances for the test sensitivities and specificities. When this proportion was zero, bacteriology and PCR were assumed independent, but this was no longer the case as this proportion increased. These eight models were compared using the Deviance Information Criterion (DIC).

#### 4.2. Second analysis

As previously, bacteriology and PCR were considered dependent tests given infection status. However, histopathology was assumed conditionally independent of the two other tests as this method analyzes morphological changes in tissues. As in the first analysis, covariances were fixed and expressed as a proportion of the maximum conditional covariances for the test sensitivities and specificities. Eight values of proportion were tested from 0.0 to 0.7 and the models were compared using the DIC. Each model was identifiable, with nine parameters to estimate (three prevalences, three sensitivities and three specificities).

#### 4.3. Bayesian computation

For all the parameters to be estimated, we used uninformative priors in the shape of a uniform distribution on the interval between 0 and 1 (modeled using the beta (1, 1) distribution). The analyses were implemented in Openbugs version 3.2.2 [17]. For each model, the first 10 000 iterations were discarded as burn-in and the next 10 000 were used for posterior inference. Two chains were run from different initial values. To assess convergence of these chains, we visually inspected the time-series plots of selected variables as well as the Gelman-Rubin diagnostic plots.

## Results

Respectively 61.7% and 22.7% of 5,211 sampled animals came from the departments of Côte d’Or and Dordogne (see Figure S2). 48.7% of them belonged to from herds without any recent history of bTB (i.e. without any declaration of herd infection between 2000 and 2012). The 5,188 animals for which age, sex and breed data were available were mainly female (81.8%) and on median 3.9 years old. Respectively 20.9% of them were less than 18 months old and 26.6% more than 6 years old. The most represented cattle breeds were Charolais (60.2% of them), Limousin (17.2%) and Prim’Holstein (6.5%).

### 1. First Analysis

Among the 5,211 animals tested by bacteriology and PCR, 2,617 (50.2%), 715 (13.7%) and 1,879 (36.1%) came from population A, B and C respectively. The cross-tabulated distribution of the outcome of the two tests (bacteriology and PCR) is shown in Table 1.

**Table 1:** Number of animals in analysis 1 according to (i) their test results for bacteriology (bac) and PCR and (ii) their population of origin.

Population	POS_bac_/POS_PCR_	POS_bac_/NEG_PCR_	NEG_bac_/POS_PCR_	NEG_bac_/NEG_PCR_	Total
A	230	29	68	2290	2617
B	29	3	12	671	715
C	52	1	9	1817	1879
Total	311	33	89	4778	5211

POS: positive/NEG: negative.

**Figure 1:**
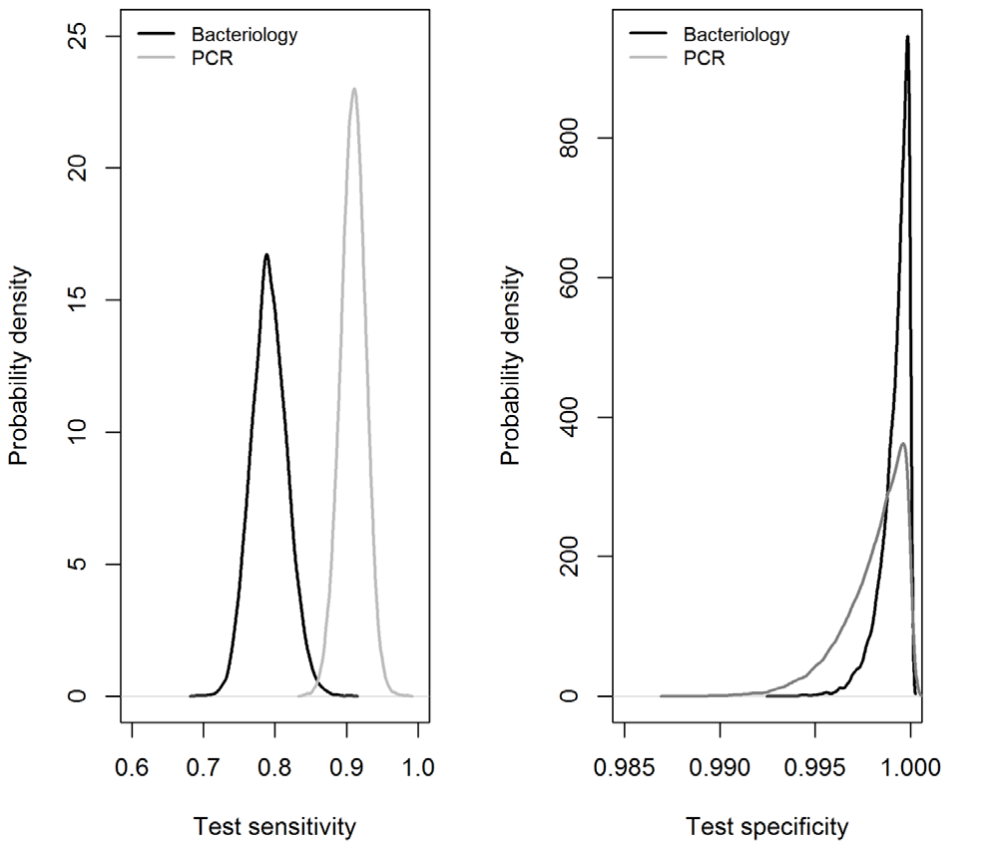
Posterior distributions for sensitivities and specificities of bacteriology and PCR. These estimations are based on the model assuming independence between bacteriology and PCR (model with the smallest DIC).

The test characteristics and infection prevalences in populations A, B and C estimated from this data are presented in Table 2. The model assuming independence between bacteriology and PCR (i.e. proportion of maximal conditional covariance equal to zero) had the smallest DIC, suggesting this model as the most fitted. However, the DIC of the eight models are very close, suggesting an equivalent fit. Bacteriology and PCR had very high estimated specificities that remained almost unchanged whatever the value of the proportion of conditional covariance between the two tests. On the contrary, the sensitivities estimates decreased as this proportion increased but sensitivity of PCR was always higher than that of bacteriology (except for models with proportions of conditional covariance ≥0.5 where posterior credible intervals of the two sensitivities overlapped; however, these models had a lower fit). Figure 1 shows the posterior distributions for sensitivities and specificities of the two tests in the model assuming independence between bacteriology and PCR (model with the smallest DIC).

**Table 2:** Mean estimates and 95% posterior credible intervals (CI) of the sensitivity (Se) and specificity (Sp) of bacteriology (bact) and PCR and population specific animal prevalences of the eight models assuming different proportions of maximum conditional covariances (analysis 1).

Conditional covariance^a^	Test characteristics (95% CI)				Prevalences (%)			DIC^b^
	Se_bact_	Sp_bact_	Se_PCR_	Sp_PCR_	A	B	C	
**0**	**79.2 (74.4–84.4)**	**99.9 (99.7–100)**	**90.9 (87.3–94.3)**	**99.8 (99.4–100.0)**	**12.5 (11.1–13.9)**	**6.1 (4.3–8.1)**	**3.3 (2.4–4.2)**	**64.94**
0.1	78.3 (73.4–83.4)	99.9 (99.6–100)	89.9 (85.9–93.7)	99.8 (99.3–100.0)	12.6 (11.2–14.1)	6.2 (4.4–8.2)	3.3 (2.5–4.2)	65.05
0.2	77.2 (72.3–82.3)	99.9 (99.6–100)	88.6 (84.1–92.8)	99.8 (99.3–100.0)	12.8 (11.3–14.3)	6.2 (4.4–8.3)	3.3 (2.5–4.3)	65.17
0.3	75.7 (70.8–81.1)	99.9 (99.6–100)	87.0 (81.9–91.9)	99.8 (99.3–100.0)	13.0 (11.5–14.6)	6.3 (4.5–8.5)	3.4 (2.5–4.4)	65.18
0.4	73.7 (68.4–79.1)	99.9 (99.6–100)	84.7 (78.6–90.5)	99.8 (99.3–100.0)	13.4 (11.7–15.1)	6.5 (4.6–8.7)	3.5 (2.5–4.5)	65.17
0.5	71.0 (65.2–77.1)	99.8 (99.4–100)	81.6 (74.4–88.5)	99.7 (99.2–100.0)	13.8 (12.1–15.7)	6.7 (4.6–9.0)	3.6 (2.6–4.7)	65.43
0.6	66.9 (59.8–73.7)	99.8 (99.3–100)	76.9 (67.7–85.4)	99.7 (99.2–100.0)	14.7 (12.5–16.9)	7.1 (4.8–9.6)	3.7 (2.6–5.0)	65.45
0.7^c^	59.8 (49.8–68.9)	99.7 (99.1–100)	68.9 (55.9–81.0)	99.6 (98.8–100.0)	16.3 (13.2–19.9)	7.8 (5.1–11.1)	4.0 (2.5–5.6)	65.66

a Proportion of maximum upper limit of conditional covariance.

b Deviance information criterion.

c For this model, a thin interval of 3 was defined in order to ensure that the MCMC chains are no longer autocorrelated.

Note: the results of the model with the lowest DIC are in bold.

### 2. Second Analysis

Among the 697 animals tested in bacteriology, histopathology and PCR, 345 (49.5%), 94 (13.5%) and 258 (37.0%) came from population A, B and C respectively. The cross-tabulated distribution of the outcome of the three tests is shown in Table 3.

**Table 3:** Number of animals in analysis 2 according to (i) their test results for bacteriology (bac), histopathology and PCR and (ii) their population of origin.

Histopathology	Population	POS_bac_/POS_PCR_	POS_bac_/NEG_PCR_	NEG_bac_/POS_PCR_	NEG_bac_/NEG_PCR_	Total
POS	A	163	14	32	31	240
	B	21	3	10	13	47
	C	32	0	2	37	71
NEG	A	8	1	9	87	105
	B	0	0	1	46	47
	C	6	0	3	178	187
Total		230	18	57	392	697

POS: positive/NEG: negative.

**Figure 2:**
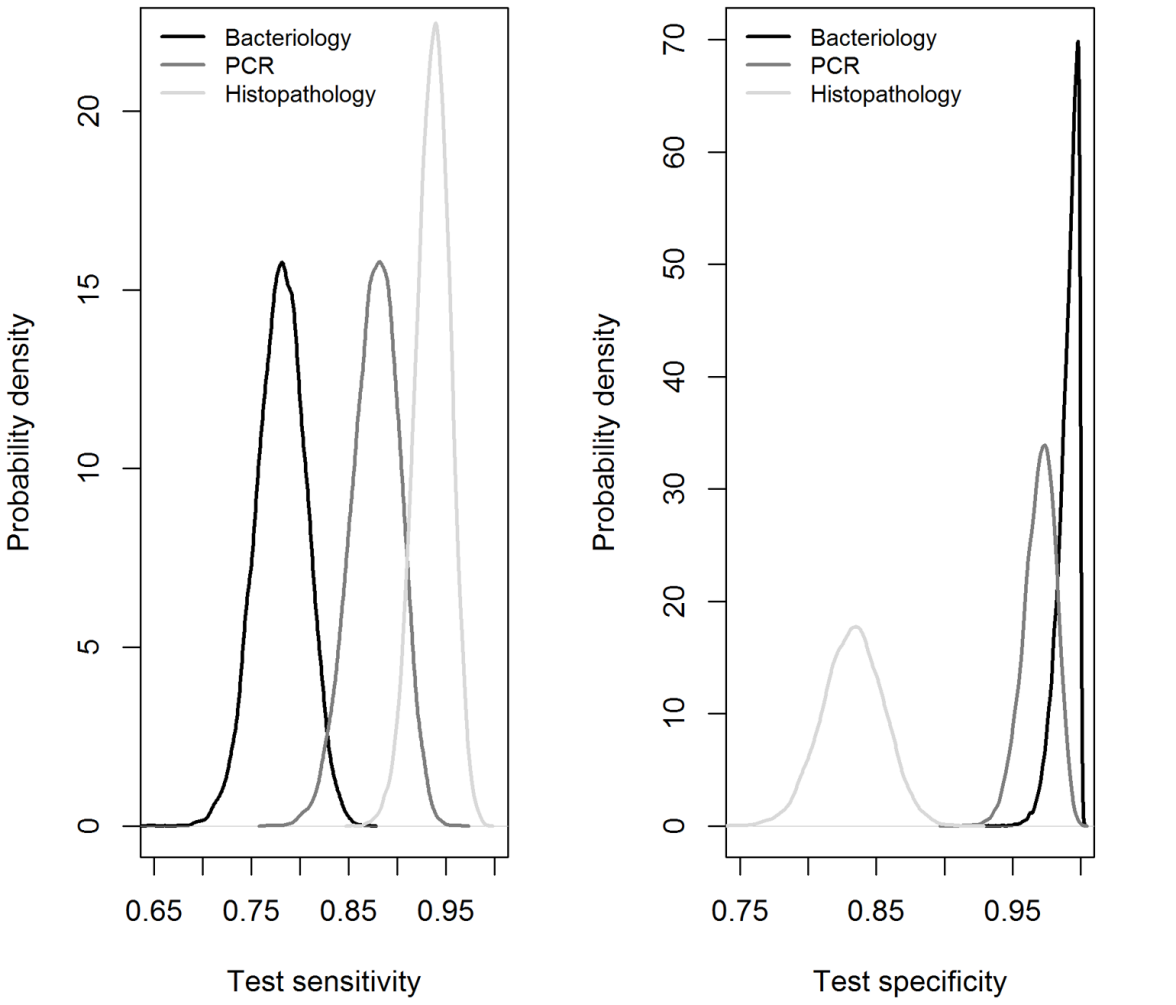
Posterior distributions for sensitivities and specificities of bacteriology, histopathology and PCR. These estimations are based on the model with a proportion of maximal conditional covariance between bacteriology and PCR equal to 0.4 (model with the smallest DIC).

The test characteristics and infection prevalences in populations A, B and C estimated from this data are presented in Table 4. The model assuming a moderate dependence between bacteriology and PCR (i.e. proportion of maximal conditional covariance equal to 0.4) had the smallest DIC, suggesting this model should be preferred. However, models with a proportion of conditional covariance from 0.3 to 0.5 had very close DIC, suggesting an equivalent fit. Specificity estimates for bacteriology and PCR decreased as the conditional covariance increased, whereas that of histopathology increased. According to the three preferred models, bacteriology and PCR had high estimated specificities. Even if their posterior 95% CI overlapped, the specificity of the bacteriology might be slightly higher than that of PCR (see Figure 2 for the model with the smallest DIC). The estimated specificity of the histopathology was much lower than those of the two other tests. Sensitivity estimates for bacteriology and PCR decreased as the conditional covariance increased, whereas the sensitivity of the histopathology remained almost unchanged. Nevertheless, sensitivity of bacteriology was always lower than that of PCR and of histopathology. Regarding the two latter tests, even if the posterior 95% CI for their sensitivities slightly overlapped, it seemed likely that sensitivity of the histopathology was higher than that of PCR (see Figure 2 for the model with the smallest DIC).

**Table 4:** Mean estimates and 95% posterior credible intervals (CI) of the sensitivity (Se) and specificity (Sp) of bacteriology, histopathology and PCR and population specific animal prevalences of the eight models assuming different proportions of maximum conditional covariances (analysis 2).

Conditional covariance^a^	Test characteristics (95% CI)						Prevalences^b^ (%)			DIC^c^
	Se_bact_	Sp_bact_	Se_hist_	Sp_hist_	Se_PCR_	Sp_PCR_	A	B	C	
0	82.2 (77.1–86.9)	99.6 (98.6–100.0)	92.9 (89.4–95.9)	80.2 (75.9–84.1)	92.3 (88.6–95.4)	97.5 (95.0–99.4)	65.3 (59.8–70.6)	36.7 (26.8–46.9)	16.0 (11.7–20.9)	116.9
0.1	81.4 (76.3–86.1)	99.6 (98.5–100.0)	93.0 (89.3–96.0)	80.8 (76.5–84.8)	91.3 (87.2–94.8)	97.4 (95.0–99.3)	66.0 (60.3–71.4)	37.1 (27.3–47.5)	16.0 (11.7–21.0)	116.2
0.2	80.5 (75.4–85.2)	99.4 (98.1–100.0)	93.2 (89.6–96.2)	81.5 (77.2–85.6)	90.2 (85.6–94.0)	97.3 (94.9–99.3)	66.8 (61.1–72.2)	37.6 (27.6–48.0)	16.0 (11.6–21.0)	115.4
0.3	79.4 (74.2–84.2)	99.3 (97.6–100.0)	93.4 (89.7–96.5)	82.4 (77.9–86.6)	88.9 (84.0–93.2)	97.2 (94.6–99.2)	67.5 (61.8–73.1)	38.3 (28.1–48.9)	16.1 (11.6–21.2)	114.8
**0.4**	**78.1 (72.9–82.8)**	**99.1 (97.1–100.0)**	**93.6 (89.9–96.9)**	**83.3 (78.7–87.6)**	**87.7 (82.5–92.3)**	**97.0 (94.3–99.0)**	**68.3 (62.5–73.9)**	**38.9 (28.5–49.8)**	**16.2 (11.5–21.4)**	**114.2**
0.5	76.6 (71.2–81.6)	98.8 (96.6–100.0)	93.9 (90.0–97.4)	84.1 (79.5–88.7)	86.5 (81.1–91.3)	96.7 (93.9–98.9)	69.0 (63.0–74.7)	39.7 (29.2–50.6)	16.3 (11.4–21.7)	114.5
0.6	75.0 (69.4–80.1)	98.5 (96.1–100.0)	94.2 (90.2–97.7)	85.1 (80.3–89.6)	85.3 (79.8–90.4)	96.3 (93.3–98.7)	69.6 (63.7–75.3)	40.6 (29.8–51.8)	16.4 (11.3–22.2)	115.8
0.7	73.5 (67.8–78.8)	98.2 (95.5–99.9)	94.6 (90.5–98.2)	85.9 (80.9–90.4)	84.6 (79.0–89.7)	95.9 (92.6–98.5)	69.9 (64.0–75.6)	41.4 (30.6–52.7)	16.5 (11.4–22.4)	119.3

a Proportion of maximum upper limit of conditional covariance.

b Analysis 2 was carried out on a subgroup of animals (animals subjected to histopathology which all presented macroscopic bTB-like lesions), hence the differences in animal prevalence estimates between analysis 1 and analysis 2.

c Deviance information criterion.

Note: the results of the model with the lowest DIC are in bold.

## Discussion

We estimated the sensitivity and specificity of bacteriology, histopathology and PCR for bTB diagnosis in the absence of a gold standard. This is, to our knowledge, the first report of a latent class analysis used for evaluation of bTB diagnostic tests. In this paper, we followed the STRADAS guidelines (Standards for Reporting of Animal Diagnostic Accuracy Studies), that were initially developed to improve the quality of reporting of the design, conduct and results of paratuberculosis test accuracy studies [18]. The analysis showed that PCR is more sensitive than bacteriology and has a good specificity (although possibly lower than that of bacteriology). This makes PCR a useful tool that could potentially become an official bTB diagnostic test within the EU. Histopathology seems to be more sensitive than bacteriology but also less specific, which means that this test cannot be used alone as a confirmation test.

Latent class analysis relies on a number of critical assumptions. First, at least two populations with differing prevalences are required. Here, the population was stratified into three subpopulations (populations A, B and C) based on the incidence of infection within each canton over the 2000–2007 period. Posterior distributions of estimated prevalences in populations A, B and C did not overlap, which suggests that the stratification of the population was successful. According to Toft et al. [19], it is preferable to have a difference in prevalence as large as possible. In our first analysis, prevalences within each population were close (11.1–13.9% in population A, 4.3–8.1% in population B and 2.4–4.2% in population C), which could affect the precision of the estimates as well as the estimates themselves [19]. However, in the second analysis, these potential biases were a priori absent as there was a very large difference in the animal prevalence estimates within population A (62.5–73.9%), B (28.5–49.8%) and C (11.5–21.4%). The second assumption is that of constant test sensitivity and specificity between populations. Johnson et al. [20] stated that a diagnostic test might have increased sensitivity when applied in high prevalence populations due to the greater burden of clinically affected animals and higher quantities of bacteria shed. There might also be problems with constant specificities across populations, e.g. because of geographic differences in cross-reactions to other mycobacteria [2]. Toft et al. [19] demonstrated that a difference in test sensitivity between populations may result in estimates that are biased toward the sensitivity of the test in the population with the highest infection prevalence. Similarly, Johnson et al. [20] showed that, when neither evaluated test is perfectly specific, the results of the Bayesian analysis are all wrong if the assumption of constant sensitivity has failed. However, in our case, bacteriology has an almost perfect specificity, which would allow us to interpret test accuracy estimates for sensitivity as estimates of the average sensitivity across populations if the assumption of constant sensitivity failed [6], [20]. Finally, bacteriology and PCR were considered as conditionally dependent tests. In the first analysis, the eight models had an equivalent DIC and the estimates were very close for models with a proportion of maximal conditional covariance from 0 to 0.3. As in Paul et al. [8], this could mean that the two tests might be conditionally dependent but this dependence does not affect sensitivity and specificity when evaluated against each other. In the second analysis, models with a proportion of maximal conditional covariance from 0.3 to 0.5 had smaller DIC, with a difference of at least 2 from the model assuming bacteriology and PCR as independent (i.e. model with a proportion of maximal conditional covariance equal to 0). Smaller DIC values indicate that these models fit better. It was therefore necessary to take into account the correlation between the two tests.

For all the parameters to be estimated, we used uninformative priors. However, bacteriology is considered to be 100% specific and either an informative prior distribution or a fixed value for this parameter could have been used in the models. We ran the model with the smallest DIC in analysis 1 and the model with the smallest DIC in analysis 2 using either an informative prior distribution (a beta (34.1664, 1.335) distribution whose mode is 99% and 5% percentile 90%) or a fixed value (100%) for the specificity of bacteriology. The estimated test characteristics and infection prevalences were influenced by none of these options (results not shown).

In this study, PCR sensitivity was higher than previously reported. Estimates of sensitivity for this test were broadly the same in the first (mean: 90.9% [87.3–94.3%]) and second analysis (mean: 87.7% [82.5–92.3%]), suggesting that PCR sensitivity was not influenced by the presence or absence of bTB-like lesions on tested animals. On the contrary, Parra et al. [21] reported a sensitivity of PCR from 61.1% for samples with non-visible lesions to 80.6% for chronic lesions when using a manual extraction system and considering bacteriology and/or presence of bTB-like lesions and positive bacterioscopy as the gold standard. In Taylor et al. [22], comparison of RD4 PCR and IS*1081* PCR with the gold standard of bacteriology showed a sensitivity of approximately 50% and 70% respectively. Thacker et al. [23] reported that out of 30 *M. bovis* culture positive tissues, only 20 were positive using IS*6110* real-time PCR and in Proaño-Perez et al. [24], eight animals were found PCR positive over the twelve positive in bacteriology. Only Cardoso et al. [25] reported a frequency of PCR-positive results similar to that of culture-positive results (51.5% versus 54.5%) over 35 lymph nodes samples from animals with macroscopic lesions consistent with *M. bovis* infection. In our study, the sensitivity of PCR was higher than that of bacteriology. It may thus be suspected that bacteriology under French conditions lacks sensitivity. The decontamination procedure of this bacteriology uses sulphuric acid (H_2_SO_4_), which is very efficient to kill contaminating microorganisms but also toxic for mycobacteria. However, the LDAR 24 and the French National Reference Laboratory produced very satisfactory results in the ring trial organized by the European Union Reference Laboratory for bTB (EURL personal communication). Thus, the assumption of poor sensitivity of French bacteriology is not justified, which is confirmed by our estimates (mean of 79.2% [74.4–84.4%] in the first analysis and of 78.1% [72.9–82.8%] in the second one). These estimates are consistent with Corner et al. [26] who reported a proportion of infected samples identified by culture between 58.0% and 80.0% depending on the culture media used and the decontamination procedure employed. It is interesting to point out that not all the PCR methods employed in the literature are equivalent (e.g. detected genome sequences are sometimes different as well DNA extraction methods). The good sensitivity reported here could then be attributable to the inherent good quality of our methodology. Besides, unlike previous studies on PCR sensitivity [21]–[25], by applying a latent class analysis, we were able to estimate the characteristics of the three tests without a gold standard. According to Toft et al. [9], when using a single, imperfect test as a reference, estimates of sensitivity and specificity will always be biased and as a result, the new test (here PCR) will always be reported to have properties which are equal to or not as good properties as those of the reference test (here bacteriology). The method we used removed these potential biases. This could also explain the discrepancy between our results and those previously reported. Bacteriology remains, however, necessary for molecular epidemiology studies. Indeed, *M. bovis* full genotyping is only possible on cloned bacterial isolates. Regarding histopathology, our estimates (mean Se of 93.6% [89.9–96.9%] and mean Sp of 83.3% [78.7–87.6%]) are consistent with those obtained by Varello et al. [5]: considering culture as a reference test on suspected lymph nodes samples from 173 cattle positive reactive in ante mortem tests, they reported a relative sensitivity of histopathology of 93.4% (95% CI: 87.4–97.1%) and a relative specificity of 92.3% (95% CI: 81.5–97.9%).

## Conclusion

This latent class analysis has provided estimates of the characteristics of two currently used tests (bacteriology and histopathology) and a novel one, a PCR method using the LSI VetMAX™ *Mycobacterium tuberculosis* Complex PCR Kit, for bTB diagnosis under field conditions in France. This study showed that PCR had a higher sensitivity than bacteriology while having a good specificity. In addition, PCR allows the confirmation of infection in less than 48 hours. Hence, PCR seems to be a good confirmatory test for bTB surveillance and control programs.

## Supporting Information

Figure S1 **Geographical location of cantons for each of the three populations.** These populations have differing expected animal infection prevalences.
(PNG)

Figure S2 **Geographical origin of the 5,211 sampled animals.**
(PNG)
