# Supplementary figures and images for: Estimation of Sensitivity and Specificity of Bacteriology, Histopathology and PCR for the Confirmatory Diagnosis of Bovine Tuberculosis Using Latent Class Analysis
Source: PLoS One. 2014 Mar 13;9(3):e90334. doi: 10.1371/journal.pone.0090334 (PMC3953111; doi:10.1371/journal.pone.0090334)

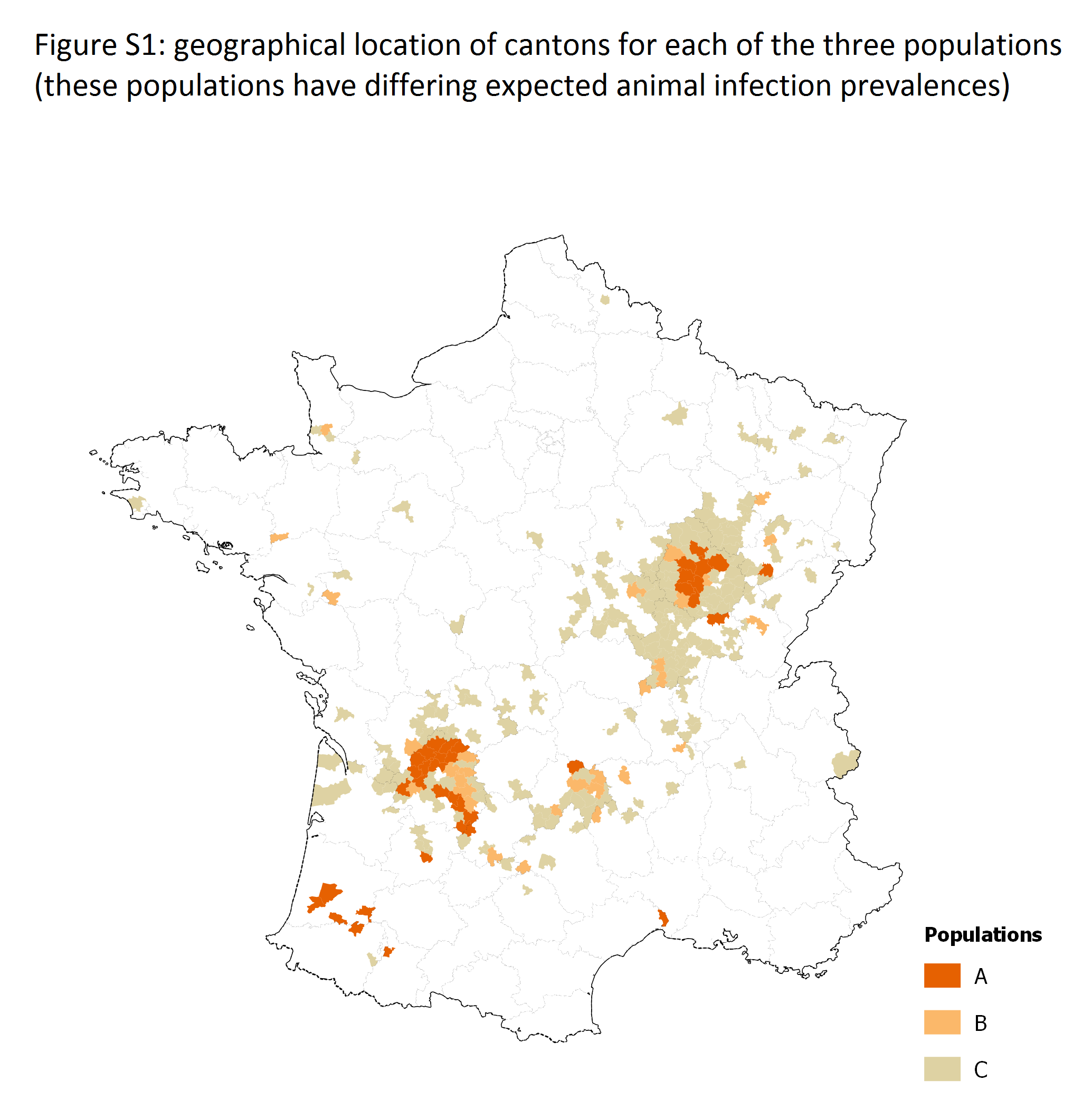

Supplement: Figure S1 — Geographical location of cantons for each of the three populations. These populations have differing expected animal infection prevalences. (PNG) [file pone.0090334.s001.png]

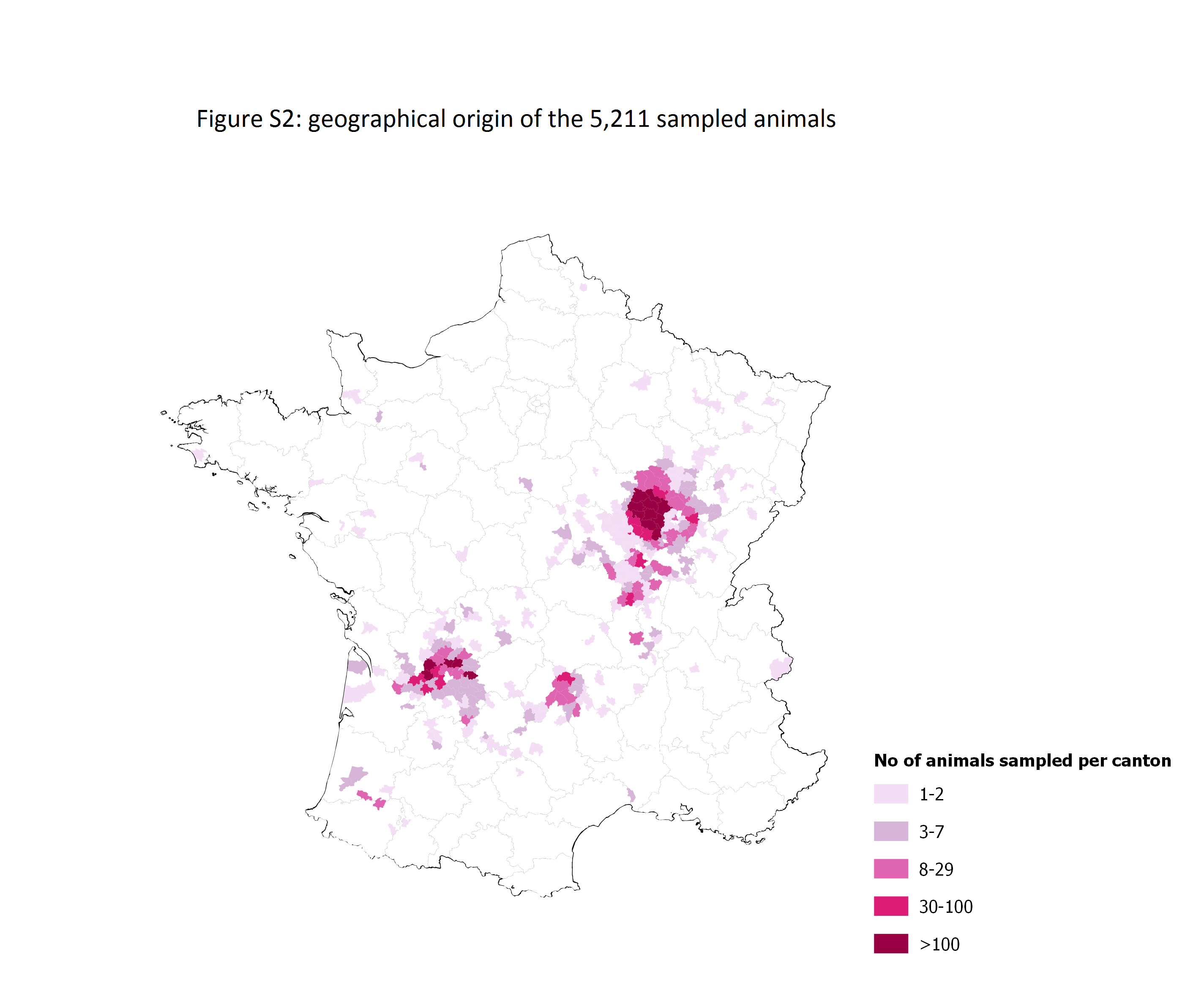

Supplement: Figure S2 — Geographical origin of the 5,211 sampled animals. (PNG) [file pone.0090334.s002.png]
